# Evaluating the Potential for Different Fabrics to Protect Grapes from Contamination by Smoke

**DOI:** 10.3390/foods14091550

**Published:** 2025-04-28

**Authors:** Tingting Shi, Renata Ristic, Kerry Wilkinson

**Affiliations:** School of Agriculture, Food and Wine, The University of Adelaide, PMB 1, Glen Osmond, SA 5064, Australia; t.shi@adelaide.edu.au (T.S.); renata.ristic@adelaide.edu.au (R.R.)

**Keywords:** activated carbon fibre, cotton, viscose, smoke taint, volatile phenols

## Abstract

Vineyard smoke exposure can lead to the accumulation of free and glycosylated volatile phenols (VPs) in grapes, negatively affecting wine quality. Activated carbon fibre (ACF) cloth has proven effective in mitigating smoke contamination of grapes, but its commercial use is hindered by low tensile strength and light transmission. This study therefore compared the efficacy of different fabrics (polyester, polypropylene, cotton and viscose) to mitigate the smoke contamination of grapes (benchmarking against ACF cloth), alongside their physical properties (i.e., tensile strength and air permeability). Polyester and polypropylene provided limited protection, whereas grapes enclosed in cotton or viscose had VP profiles that were comparable to grapes enclosed in ACF cloth (i.e., VP concentrations ≤ 5.3 µg/kg). In a subsequent trial, ACF cloth prevented the uptake of >90% of smoke-derived VPs during ten successive smoke treatments, but after repeated smoke exposure, VP concentrations had increased in grapes enclosed in cotton and viscose, presumably due to saturation. Washing and drying restored the protection afforded by cotton and viscose but resulted in the disintegration of the ACF cloth. However, the application of a non-woven fabric to one or both sides of the ACF cloth improved tensile strength, without significantly compromising air permeability. These findings demonstrate the potential for fabric coverings to be used to mitigate the occurrence of smoke taint in the vineyard, with ACF affording superior protection.

## 1. Introduction

Bushfires continue to occur, with greater frequency and intensity, in wine-producing regions around the world, resulting in substantial economic losses due to vineyard smoke exposure [1]. When grapevines are exposed to smoke, volatile phenols (VPs) such as guaiacol, 4-methylguaiacol, *o*-, *m*- and *p*-cresols, syringol and 4-methylsyringol are absorbed by berries, and are subsequently glycosylated, accumulating as non-volatile glycoconjugates [2,3,4,5,6,7]. During fermentation, these glycoconjugates can be hydrolysed, releasing free VPs into wine [8,9,10]. Both free and glycosylated VPs can impart undesirable smoky, burnt rubber, and ashy characters to wine [11,12,13,14]. This phenomenon, known as ‘smoke taint’, compromises wine quality, reduces marketability and negatively impacts consumer acceptance [15].

Various strategies have been explored to mitigate the intensity of smoke taint in grapes and wine. Once smoke exposure of grapes occurs, harvesting and processing decisions can influence the extraction of free and glycosylated VPs; hand-harvesting, whole-bunch pressing and pressing at lower fruit temperatures (10 °C) and juice extraction rates (<400 L/t) have therefore been recommended [16,17,18]. Winemaking interventions, such as limiting skin contact time, yeast strain selection and the use of oak and tannin additions, can influence the sensory perception of smoke taint in wine [9,10,19] but do not remove smoke-derived VPs, so efficacy is limited [20]. In contrast, remedial treatments involving the addition of adsorbent materials (e.g., activated carbon, molecularly imprinted polymers and adsorbent or ion exchange resins) to juice, must or wine can remove free and glycosylated VPs to varying degrees [21,22,23,24,25,26]. These treatments can either be applied directly or used in combination with membrane filtration [21,26] or spinning cone column distillation [24] to minimise the removal of desirable aroma, flavour or colour attributes. Nevertheless, vineyard-based strategies that prevent smoke contamination of grapes in the first place are still preferable.

Amongst the vineyard-based approaches to the mitigation of fruit exposure to smoke, protective treatments such as in-canopy misting [7] and spray applications of kaolin [27,28], biofilms [29] and edible coatings [30] have been evaluated. However, the efficacy of these mitigation strategies remains questionable, largely due to challenges associated with achieving uniform coverage of fruit and/or timely application of sprays prior to grapevine smoke exposure. Two recent studies demonstrated the potential for activated carbon fibre (ACF) cloth to prevent the smoke contamination of grapes [31,32]. Enclosing grapes in ACF cloth prior to smoke exposure prevented significant uptake of VPs—even during exposure to dense smoke [32]—likely due to the high surface area and excellent adsorption capacity of ACF cloth [33].

Historically, the high porosity and adsorptive properties of ACF cloth have been exploited in applications including air purification and personal protective clothing [34,35,36]. The production of ACF cloth involves two steps. Initially, a carbon-rich precursor—typically rayon (viscose) or another fabric made from regenerated cellulose—is subjected to carbonisation, i.e., thermal degradation at high temperatures (~600–1000 °C) in an inert atmosphere [33]. This removes non-carbon elements and yields a basic carbon structure. Subsequently, the pyrolysed carbon fibres undergo activation using carbon dioxide or steam, a process that creates an extensive network of micropores, dramatically increasing surface area [33,36]. However, the activation process compromises the structural integrity (i.e., the flexibility and strength) of ACF [37], and the decreased tensile strength makes ACF cloth more prone to tearing [33]. The carbonisation process also renders ACF cloth black in colour, which reduces light transmission. This is problematic in the vineyard given that the application of black material to grapevines would lead to shading, which in turn could cause premature leaf senescence, adversely affecting berry ripening [38,39,40].

The use of a fabric that can offer protection against smoke contamination, with improved mechanical durability (tensile strength) and light transmission would help overcome shortcomings associated with the use of ACF cloth. This study therefore compared the extent to which different fabrics (polyester, polypropylene, cotton and viscose) could mitigate the absorption of smoke-derived VPs by grapes, as a measure of smoke taint, benchmarking against the performance of ACF cloth. The physical properties of fabrics (i.e., tensile strength and air permeability) were also compared, along with practical considerations, such as the ability for fabrics to be washed and reused.

## 2. Materials and Methods

### 2.1. Preparation of Fabric Coverings

Activated carbon fibre (ACF) fabrics were sourced from Nature Technologies (Hangzhou, China), while polyester, polypropylene, cotton and viscose fabrics (white in colour) were purchased from Lincraft (Adelaide, SA, Australia), Ferrier Fashion Fabrics (Fullarton, SA, Australia) and the Fabric Store (Auckland, New Zealand). Fabrics were selected (with input from a textile expert) to ensure the inclusion of common natural (cotton) and synthetic (polyester and polypropylene) materials, with viscose included as the base material from which ACF is made. Rectangular pieces of each fabric (~30 × 60 cm) were folded in half and the two adjacent sides were stitched together to make the fabric coverings used in smoke exposure trials, with newly made coverings used for each trial unless otherwise specified.

### 2.2. Smoke Exposure Trials

A series of trials were undertaken to evaluate to what extent different fabrics could prevent the smoke contamination of grapes. In each case, this involved enclosing a bunch of mature grapes (total soluble solids were 22–23° Brix; cv. Merlot or Viognier, depending on availability, harvested from vineyards located at the University of Adelaide’s Waite Campus in Urrbrae, South Australia (34°58′ S, 138°38′ E)) in different fabric bags (in triplicate), which were then suspended on a rack in a purpose-built smoke chamber (as described previously [32]). One replicate from each fabric treatment, along with a smoke-only treatment (i.e., a bunch of grapes with no fabric covering), were randomly positioned on the top, middle and bottom tiers of the rack. Smoke was generated by combusting 100 g of barley straw. Immediately after smoke exposure (for 15 min), grapes (50 berries per bunch per treatment per replicate, chosen randomly) were collected, homogenised using a T18 Ultra Turrax (IKA, Staufen, Germany) and frozen at –4 °C prior to compositional analysis. Grapes were also collected (in triplicate) from bunches that were not exposed to smoke as controls.

#### 2.2.1. Trial 1: Evaluation of Different Fabrics During Single Smoke Exposure

A preliminary trial was undertaken to compare the uptake of smoke-derived VPs by Merlot grapes enclosed in different fabrics (ACF cloth, polyester, polypropylene, cotton and viscose) during a single smoke application (15 min).

#### 2.2.2. Trial 2: Evaluation of Different Fabrics During Repeated Smoke Exposure

Based on results from the preliminary trial, a trial involving repeated smoke exposure of Viognier grapes was undertaken. Grape bunches were again enclosed in fabric coverings (made from the ACF cloth, two kinds of cotton and three kinds of viscose) prior to smoke exposure. Whereas the same fabric coverings were used for each of the successive smoke treatments (10 × 15 min), grapes were replaced between each application of smoke. Fresh bunches of grapes were also used for successive smoke-only treatments (i.e., the treatment involving smoke exposure of grapes with no fabric covering).

#### 2.2.3. Trial 3: Evaluation of Fabric Re-Usability

Following the repeated smoke exposure trial, fabric coverings were turned inside out and fresh bunches of Viognier grapes were placed in each before they were stored at ambient temperature (23 °C) for 72 h. After grapes were sampled for compositional analysis (measuring both free and glycosylated VPs), the bags were turned inside out again (i.e., back to their original form) and rinsed, machine-washed and air-dried. An additional smoke exposure trial (15 min) was then undertaken to compare the uptake of smoke-derived VPs by Merlot grapes enclosed in the washed fabric bags. However, the ACF cloth coverings could not be re-used as they disintegrated during the washing and drying process.

#### 2.2.4. Trial 4: Evaluation of Reinforced Activated Carbon Fibre Cloth

A final smoke exposure trial (15 min) was conducted to compare the uptake of smoke-derived VPs by Merlot grapes enclosed in coverings made from the ACF cloth, and the same ACF cloth bonded with a non-woven fabric on one or both sides (Figure S1).

### 2.3. Compositional Analysis of Grapes

The concentration of VPs (i.e., guaiacol, 4-methylguaiacol, *o*-, *m*- and *p*-cresol, syringol and 4-methylsyringol) were measured in grape homogenate as chemical markers of smoke taint using an Agilent 6890 gas chromatograph coupled to a 5973 mass spectrometer (Agilent Technologies, Forest Hill, VIC, Australia) and established stable isotope dilution assays [41,42]. Internal standards (d_4_-guaiacol, d_3_-syringol and d_5_-*o*-cresol) were sourced from LGC Standards (Petaluma, CA, USA). Sample preparation and instrument operating conditions were as previously reported [41,42]. ChemStation (version E.02.00.493) and MassHunter (version B.09.00) software were used for data acquisition and processing, respectively. The limits of quantitation for each VP were 1 µg/kg.

The concentration of glycoconjugates of VPs (i.e., glucosides, pentose glucosides, gentiobiosides and rutinosides) were also measured in selected grape homogenate samples (as syringol gentiobioside equivalents) using an Agilent 1200 high-performance liquid chromatograph equipped with a 1290 binary pump and coupled to an AB SCIEX Triple Quad^TM^ 4500 tandem mass spectrometer (Agilent Technologies), with a Turbo V^TM^ ion source (Framingham, MA, USA), and established stable isotope dilution assays [42]. The internal standard (d_3_-syringol gentiobioside) was again sourced from LGC Standards. Sample preparation and instrument operating conditions were as previously reported [42], and the limits of quantitation were 1 µg/kg. SCIEX software (version 1.7.0.36606) was used for data analysis.

### 2.4. Physical Testing of Fabrics

Fabric thickness was measured using a Mitutoyo 500-196-30 digital calliper (West Heidelberg, VIC, Australia), with triplicate measures taken for one bag of each type of fabric. Samples of each fabric were also sent to the Australian Wool Testing Authority Ltd. (Flemington, VIC, Australia), an accredited materials testing laboratory, for physical testing. Air permeability and tensile strength (breaking force) were measured according to Australian Standards 2001.2.34 [43] and 2001.2.3.1 [44], respectively.

### 2.5. Statistical Analysis

Statistical analysis of compositional data was performed using XLSTAT (version 2023, Lumivero, Denver, CO, USA). One-way analysis of variance (ANOVA) was applied to determine statistically significant treatment effects, with differences between means determined by HSD post hoc tests at *p* < 0.05.

## 3. Results and Discussion

### 3.1. Evaluation of Different Fabrics During Single Smoke Exposure

A preliminary trial was undertaken to compare the extent to which different fabrics—polyester, polypropylene, cotton and viscose (i.e., two synthetic textiles, a natural textile and a semi-synthetic textile)—could protect grapes from smoke contamination, benchmarking performance against the ACF cloth used in previous studies [31,32].

None of the VPs measured as markers of smoke taint were detected in control grapes, but they were detected at 2–36 µg/kg in grapes that were not enclosed in fabric coverings during smoke exposure (Table 1). Amongst the different fabric coverings, the ACF cloth afforded the greatest protection; 4-methylguaiacol, *p*-cresol, syringol and 4-methylsyringol were not detected in grapes enclosed in ACF cloth, while guaiacol, and *o*- and *m*-cresol were observed at 1.3–4.3 µg/kg. Cotton and viscose also performed well, with grapes enclosed in these fabrics containing only 1.0–5.3 µg/kg of guaiacol, 4-methylguaiacol, and *o*- and *m*-cresol; the presence of 4-methylguaiacol was the only significant compositional difference observed compared with grapes enclosed in ACF cloth. In contrast, polyester and polypropylene coverings provided only partial protection, with significantly higher concentrations of all VPs (except 4-methylsyringol) observed in grapes enclosed in these fabrics (Table 1).

The physical properties, including tensile strength, of the different fabrics are shown in Table 2. The performance of synthetic textiles may in part reflect fabric thickness and air permeability, given that the polyester was considerably thinner than the other fabrics (0.064 mm, compared with 0.182 to 0.457 mm), while the air permeability of polypropylene exceeded that of the other fabrics evaluated in the preliminary trial, as well as the maximum flow rate (680 cm^3^/cm^2^.s) of the test method. However, previous research has shown that the structure and chemical properties of different textiles affect their capacity to absorb (cigarette) smoke [45]. Natural fibres, such as cotton and wool, have large, irregular surfaces and a porous structure [46], whereas synthetic fibres have smoother, more uniform surfaces [47], such that natural fibres were found to absorb more (cigarette) smoke than synthetic materials [45,48]. Although the synthetic fabrics evaluated in the current study did partially mitigate the uptake of some VPs (4-methylguaiacol, *m*-cresol, and syringol, in particular), they were not included in subsequent trials due to their limited efficacy relative to the ACF cloth, cotton and viscose.

### 3.2. Evaluation of Different Fabrics During Repeated Smoke Exposure

**Figure 1:**
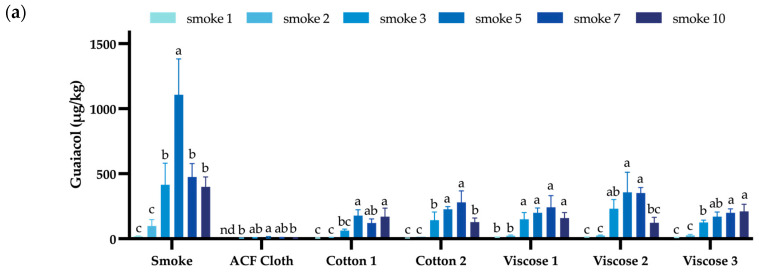

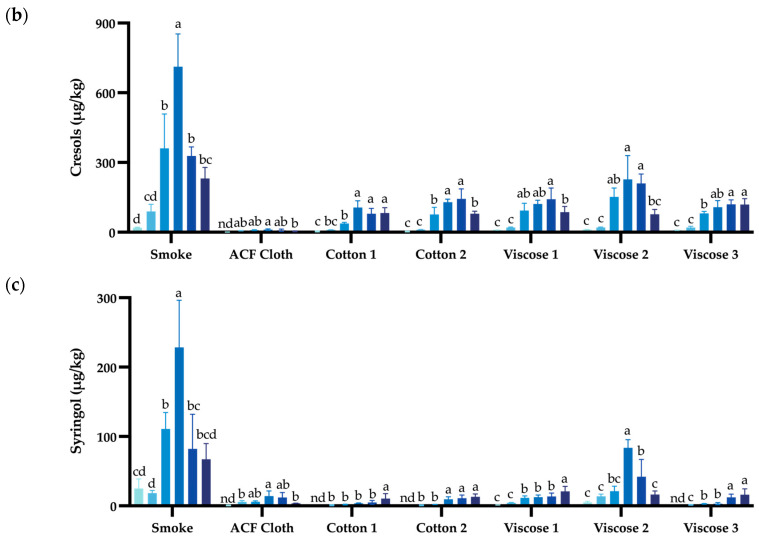
Concentration of (**a**) guaiacol, (**b**) cresols and (**c**) syringol (µg/kg) in control and smoke-exposed Viognier grapes, with and without bunches being enclosed in ACF cloth, cotton or viscose coverings during the first, second, third, fifth, seventh and tenth replicate smoke treatments. Values are means of three replicates (*n* = 3) ± standard deviation. Different letters (by fabric type) indicate significant differences between smoke treatments (*p* < 0.05, one-way ANOVA); nd = not detected.

The protection afforded by two cotton and three viscose fabrics was evaluated via a trial involving repeated exposure to smoke (10 × 15 min), again benchmarking against the ACF cloth. Volatile phenols were not detected in control (unsmoked) grapes, while grapes that were not enclosed in fabric coverings during smoke exposure consistently yielded the highest VP concentrations (Figure 1, Table S1). However, the VP profiles of the smoke-exposed grapes varied significantly between replicate smoke treatments. Despite standardising the mass of fuel being combusted (100 g of barley straw) and the duration of smoke exposure (15 min), wind affected the quantity of smoke transferred from the smoker to the smoke chamber during the first two replicate smoke treatments. This resulted in grapes being exposed to less dense smoke, and thus, lower levels of grape VPs (Figure 1, Table S1), in agreement with previous research that demonstrated that smoke density affects the uptake of VPs by grapes [49]. This nevertheless provided an opportunity to compare fabric performance under varying degrees of smoke density.

The ACF cloth consistently provided superior protection of fruit from smoke contamination, yielding the lowest grape VP concentrations across replicate exposures (Figure 1, Table S1). Volatile phenols were not detected in grapes enclosed in ACF cloth following the first smoke treatment, and thereafter, total grape VPs ranged from ~13 to 41 µg/kg. Even during exposure to dense smoke (e.g., the fifth replicate smoke treatment), ACF cloth prevented the uptake of >90% of the total VPs observed in smoke-exposed grapes, consistent with previous findings [31]. Interestingly, whereas guaiacol and *o*-cresol were typically the two most abundant VPs in smoke-exposed grapes (Table S1), syringol was detected at concentrations comparable to or higher than guaiacol in grapes enclosed in ACF cloth, which might indicate some variability in the sorptive affinity of the ACF cloth towards different VPs.

The cotton and viscose fabrics also protected grapes from smoke contamination, just not to the same extent as the ACF cloth, especially during the higher density and/or later smoke treatments (Figure 1, Table S1). Cotton 1 (the same cotton fabric used in the preliminary trial) initially performed well, yielding grape VP concentrations comparable to those of grapes enclosed in ACF cloth during the first three smoke treatments. However, significantly higher guaiacol, total cresol and syringol concentrations were observed in grapes enclosed in Cotton 1 during subsequent smoke treatments (Figure 1), indicating decreased efficacy, potentially due to saturation. Nevertheless, Cotton 1 still prevented the uptake of >60% of the VPs detected in smoke-exposed grapes after the tenth successive smoke treatment.

Similar results were observed for Cotton 2, the lighter weight (thinner) cotton fabric (Table 2). The protection afforded to grapes during the initial smoke treatments declined when more dense smoke treatments were applied, yielding grapes with significantly higher VP concentrations than the grapes that were enclosed in ACF cloth during smoke exposure (Figure 1). However, with the exception of the seventh smoke treatment, the VP profiles of grapes enclosed in the two different cotton fabrics were not significantly different following each replicate smoke treatment (Table S1). Despite some evidence of saturation (Figure 1c), Cotton 2 prevented the uptake of >50% of the total VPs observed in smoke-exposed grapes after the tenth successive smoke treatment (Figure 1, Table S1). This was attributed to the fabric absorption of smoke-derived VPs, analogous to the absorption of (cigarette) smoke and associated volatile compounds by cotton (and other textiles) reported previously [45,48,50].

The performance of the viscose fabrics was generally comparable to that of the cotton fabrics (Figure 1, Table S1). Some significant differences were observed following the first two less dense smoke treatments (Table S1), but there were fewer statistically significant differences in VP profiles following subsequent smoke applications. Amongst the grapes that were enclosed in fabric coverings during smoke exposure, the lighter weight (thinner) Viscose 2 (Table 2) tended to yield the highest VP concentrations, and thus, was the fabric that provided the least protection from smoke contamination (Table S1). Grape VP profiles for Viscose 2 most closely mirrored the pattern of VP uptake in smoke-exposed grapes (Figure 1), suggesting the thinner fabric had lower sorptive capacity; there was no evidence of saturation for Viscose 2, whereas saturation looked to have occurred in Viscose 3, and to a lesser extent in Viscose 1, following the ten successive smoke treatments (Figure 1). Nevertheless, the viscose fabrics still prevented the uptake of ~50–70% of the VPs detected in smoke-exposed grapes after the tenth replicate smoke treatment (Table S1).

The relative performance of cotton and viscose fabrics may, again, in part reflect differences in fabric thickness and/or air permeability (Table 2), but findings from previous studies suggest cotton and viscose exhibit distinct absorption behaviours towards the volatile organic compounds (VOCs) present in cigarette smoke, which were attributed to differences in fabric structure and chemical properties [45,51,52]. In the case of the ACF cloth, the superior protection/performance is derived from the high surface area and adsorption properties afforded by the activation process employed during production [33,36]. However, as indicated above, this comes at a cost to structural integrity, such that the ACF cloth had significantly reduced tensile strength compared to the other fabrics (Table 2).

### 3.3. Evaluation of Fabric Re-Usability

To evaluate the potential for fabric coverings to be re-used, thereby improving their functionality, trials were undertaken to determine to what extent (i) the desorption of VPs from smoke-exposed fabrics could contaminate grapes and (ii) washing smoke-exposed fabrics could overcome saturation to restore sorptive capacity.

#### 3.3.1. Desorption of Volatile Phenols from Smoke-Exposed Fabrics

The analysis of grapes following their enclosure in fabric coverings that had been repeatedly exposed to smoke (and turned inside out) demonstrated that significant quantities of VPs were indeed desorbed from the fabrics and subsequently absorbed by fruit (Table 3). This was not surprising given that various textiles, including cotton, have previously been shown to sequester and later emit smoke-derived VOCs [50,53]. However, the extent to which grapes were contaminated by different VPs varied considerably between fabrics. Grapes enclosed in Cotton 1 and Viscose 2 contained significantly higher levels of *m*-cresol, while grapes enclosed in ACF cloth had the highest concentrations of syringol (suggesting that the sorptive affinity of ACF cloth towards syringol may be lower than for other VPs). Statistical analysis (ANOVA) suggested that the differences observed in the concentration of other grape VPs were not significant at *p* < 0.05. However, if *p*-values were relaxed to <0.1, then the differences in guaiacol, and *o*- and *p*-cresol were also significant, with higher concentrations generally being observed for grapes enclosed in Cotton 1 and Viscose 1, while lower levels were observed for grapes enclosed in ACF cloth and Viscose 2 (Table 3).

Because grapes were enclosed in smoke-exposed fabrics for 72 h, VPs were detected in both free and glycosylated forms (Table 3 and Table 4), i.e., following desorption from fabric and then absorption by grapes, in vivo glycosylation of VPs occurred, as has been reported for smoke-exposed excised bunches in numerous previous studies [29,32,54,55]. Low levels of glycosylated VPs were detected in control (unsmoked) grapes, i.e., ≤24 µg/kg, whereas for some smoke-exposed fabrics, grape VP glycoside levels were several hundred µg/kg (Table 4 and Table S2). The highest concentrations of glycosylated VPs were generally observed in grapes corresponding to Cotton 1 and 2 and Viscose 1 and 3. The lower levels of glycosylated VPs detected in grapes corresponding to ACF cloth suggest VPs may have been desorbed from ACF cloth at lower rates than occurred for other smoke-exposed fabrics. In contrast, the lower glycosylated VPs observed in grapes corresponding to the lighter weight (thinner) Viscose 2 might reflect less absorption of VPs relative to heavier (thicker) fabrics.

The relatively high levels of free VPs observed in grapes suggest desorption was still occurring at the end of the trial, i.e., after 72 h of exposure of grapes to smoke-exposed fabrics. Differences in VP profiles (both relative abundances and the distribution of free vs. glycosylated VPs) likely reflect differences in the kinetics of their desorption by fabrics, subsequent absorption by grapes and possibly also re-adsorption by fabrics. Fabric surface structure and porosity, along with the boiling point, vapour pressure and polarity of VOCs, are known to affect the rates of sorption and desorption [56,57]. Regardless, these results demonstrate the potential risk for secondary contamination of fruit where smoke-exposed fabrics are re-applied, not only to bunches of grape as coverings, as occurred in the current study, but also to grapevines—i.e., when enclosing the fruit zone of a grapevine, as might reasonably be expected to occur in a commercial setting—especially where the smoke-exposed fabric surface comes into contact with or close proximity to fruit.

#### 3.3.2. Performance of Different Smoke-Exposed Fabrics After Washing

Following the desorption trial, the smoke-exposed fabrics were washed and air-dried, for use in an additional smoke-exposure trial that sought to evaluate whether laundering could restore the sorptive capacity of the different fabrics. Prior to being washed, the smoke-exposed fabrics smelled highly smoky, but smoke aromas were no longer apparent for cotton and viscose fabrics after washing. An attempt to wash the ACF cloth resulted in its disintegration, such that it could not be used in the additional smoke trial.

The VP profiles of grapes obtained from the first replicate smoke treatment (Section 3.2) and the smoke treatment applied after smoke-exposed fabrics were washed are compared in Table 5. Similar VP concentrations were observed for smoke-exposed grapes (i.e., grapes that were not enclosed in fabric coverings), indicating that comparable levels of smoke exposure were achieved. Grapes enclosed in washed smoke-exposed fabrics generally contained similar or slightly higher VP levels than grapes that were enclosed in the same fabrics for the first time. This suggests that laundering removed much of the smoke residue acquired during repeated smoke exposure, such that sorptive capacity was significantly (but not fully) restored. These results agreed with previous studies that found laundering textiles can affect their structural integrity and functional properties (including thermal and/or chemical protection) [58,59,60].

Collectively, these results demonstrate that cotton and viscose coverings could be re-used, but they would need to be appropriately maintained, stored and reapplied to ensure ongoing functionality and avoid secondary contamination. In the case of ACF cloth, the superior sorptive capacity (relative to other fabrics) enables its re-use without washing, but care needs to be taken during handling and application to prevent damage (e.g., tearing).

### 3.4. Evaluation of Reinforced Activated Carbon Fibre Cloth

A key limitation of the ACF cloth is its low tensile strength, which compromises its durability, and thus, functionality. To overcome this shortcoming, a final trial was undertaken to evaluate the performance of the ACF cloth following heat-based bonding with a non-woven fabric on one or both sides (Figure S1).

The tensile strength of the ACF fabric was substantially improved with the inclusion of single- or double-sided backing (Table 2). The breaking force increased 2.0- and 3.8-fold lengthways and 1.4- and 2.2-fold widthways, with single- and double-backing, respectively. Nevertheless, breaking forces were still well below those measured for the different cotton and viscose fabrics (Table 2). Whereas the single backing had little impact on fabric thickness or air permeability, the ACF cloth with double backing was heavier, i.e., thickness increased ~38%, resulting in a modest decrease in air permeability (Table 2). Even so, the air permeability of the double-backed ACF cloth still exceeded that of the cotton fabrics and Viscose 3. In a commercial vineyard, the combination of black fabric—which converts light energy into heat energy more efficiently than white fabric—and decreased air permeability could have significant implications for the grapevine microclimate, and therefore vine and fruit physiology. The intensity of light, and to a lesser degree, the quality of light (i.e., spectral distribution), are critical for berry development, sugar accumulation and grape phenolic and flavour profiles [61,62]. Shading, particularly in cool climates, can therefore be detrimental to grape and wine quality [63,64]. Furthermore, disease pressure can increase when shading nets are used due to the combined effects of increased canopy temperature and/or humidity, and reduced airflow [40,65].

As before, VPs were not detected in control (unsmoked) grapes, but they were present at 3.4–56.9 µg/kg in grapes that were not enclosed in ACF cloth during smoke exposure, and at significantly lower levels, ≤7.8 µg/kg, when grapes were enclosed in ACF cloth during smoke exposure (Table 6). In comparison, only 1.6 and 2.1 µg/kg of syringol were detected in grapes enclosed in re-enforced ACF cloth. These findings suggest that the increased thickness of the bonded ACF cloth enhanced VP adsorption and/or barrier efficiency. The persistence of low levels of syringol in grapes again suggests differences in the sorptive affinity of ACF cloth amongst VPs, warranting further investigation into the binding selectivity of ACF cloth.

## 4. Conclusions

The extent to which different fabrics can mitigate the absorption of smoke-derived VPs by grapes, thereby preventing smoke taint, was evaluated via a series of experiments simulating smoke exposure using a purpose-built smoke chamber. Whilst the use of a model system does not exactly replicate the conditions under which smoke exposure occurs in a commercial vineyard, it affords an efficient and convenient approach to smoke taint research, enabling excised bunches of grapes to be exposed to smoke of sufficient density to elicit detectable levels of taint in short periods of time (i.e., 15 min). Using the model system, synthetic materials (i.e., polyester and polypropylene) were shown to provide limited protection, whereas grapes enclosed in cotton or viscose had VP profiles that were comparable to grapes enclosed in ACF cloth. While the ACF cloth continued to mitigate the uptake of VPs during repeated smoke applications, increased VP concentrations were observed in grapes enclosed in cotton and viscose, suggesting saturation. Laundering smoke-exposed cotton and viscose restored the sorptive capacity of these fabrics, enabling their reuse. Although the standard ACF cloth provides excellent protection against smoke contamination of grapes, it is prone to tearing. However, bonding the ACF cloth to a non-woven fabric was found to significantly improve tensile strength. The extent to which challenges associated with light transmission, heat transfer and airflow (i.e., grapevine microclimate and fruit physiology), as well as durability and reusability, can be overcome by using bonded ACF cloth, cotton or viscose, will be the subject of future studies. Field trials evaluating the performance of different fabrics in a vineyard setting, including how readily and effectively the grapevine fruit zone can be enclosed in each fabric, are needed to establish their true commercial viability. Technoeconomic analysis should also be performed to assess the relative cost of applying the different fabrics to grapevines (noting that standard ACF cloth is several times more expensive than cotton or viscose) vs. the financial loss that would be incurred as a consequence of having no protection from smoke taint due to bushfires.

## Figures and Tables

**Table 1:** Concentration of volatile phenols (µg/kg) in control and smoke-exposed Merlot grapes, with and without bunches being enclosed in different fabric coverings during smoke exposure.

	Guaiacol	4-Methyl Guaiacol	*o*-Cresol	*m*-Cresol	*p*-Cresol	Syringol	4-Methyl Syringol
control	nd	nd	nd	nd	nd	nd	nd
smoke	16.0 ± 0.1 a	5.0 ± 0.1 a	7.5 ± 0.5 a	7.0 ± 0.1 a	2.0 ± 0.1	36.0 ± 0.1 a	7.0 ± 0.1
ACF cloth	4.3 ± 1.2 c	nd	1.7 ± 0.3 c	1.3 ± 0.7 c	nd	nd	nd
polyester	14.3 ± 2.6 ab	3.7 ± 0.7 b	6.0 ± 1.2 ab	4.0 ± 0.6 b	1.0 ± 0.6	5.0 ± 0.6 b	nd
polypropylene	10.7 ± 1.2 b	2.3 ± 0.3 bc	5.3 ± 0.3 b	3.7 ± 0.7 b	1.0 ± 0.6	6.0 ± 1.0 b	nd
cotton	5.3 ± 0.7 c	1.3 ± 0.3 c	2.7 ± 0.3 c	1.3 ± 0.3 c	nd	nd	nd
viscose	5.0 ± 0.1 c	1.3 ± 0.3 c	2.0 ± 0.1 c	1.0 ± 0.1 c	nd	nd	nd
*p*	<0.001	<0.001	<0.001	<0.001	0.165	<0.001	na

Values are means of three replicates (*n* = 3) ± standard deviation; nd = not detected. Different letters (within columns) indicate significant differences amongst treatments (*p* < 0.05, one-way ANOVA); na = not applicable.

**Table 2:** Physical properties (thickness, air permeability and breaking force) of different fabrics.

	Thickness (mm)	Air Permeability ^1^ (cm^3^/cm^2^.s)	Max. Force Length (N/50 mm)	Max. Force ^2^ Width (N/50 mm)	Elongation at Max. Force ^2^ Length (%)	Elongation at Max. Force ^2^ Width (%)
ACF cloth *	0.332	44.9 and 42.4	15	10	1.4	8.5
polyester *	0.064	30.6 and 31.0	450	470	28.5	30.5
polypropylene *	0.225	>680	58	na	119	na
cotton 1 *	0.415	22.4 and 22.7	690	300	19.0	15.5
cotton 2	0.182	18.1 and 17.7	720	300	11.0	13.5
viscose 1 *	0.215	37.2 and 37.4	340	390	34.0	30.5
viscose 2	0.182	>680	100	na	44.0	na
viscose 3	0.250	23.5 and 23.2	520	320	16.0	29.5
ACF (single backing)	0.345	46.2 and 45.6	30	14	6.4	16.0
ACF (double backing)	0.457	33.0 and 33.6	57	22	8.0	17.0

^1^ Permeability of fabric to air [43] (facing towards and away from airflow) measured at 20 ± 5 °C and 65 ± 5% relative humidity; maximum flow rate of apparatus was 680 cm^3^/cm^2^.s; *n* = 20 replicates; surface area tested = 5.08 cm^2^. ^2^ Maximum force and elongation at maximum force [44] (lengthways and widthways); *n* = 5 replicates; na = not available (due to insufficient sample or the nature of fabric construction). * indicates fabrics evaluated in the preliminary trial.

**Table 3:** Concentration of volatile phenols (µg/kg) detected in Viognier grapes that were enclosed in different fabric coverings (for 72 h), that had been turned inside out following repeated smoke exposure.

	Guaiacol	4-Methyl Guaiacol	*o*-Cresol	*m*-Cresol	*p*-Cresol	Syringol	4-Methyl Syringol
ACF cloth *	35.7 ± 10.9 b	6.6 ± 2.0	11.7 ± 2.5 c	11.4 ± 3.0 b	12.2 ± 3.2 b	52.4 ± 20.9 a	3.0 ± 0.7
cotton 1 *	269 ± 178 a	21.2 ± 13.6	146 ± 97.9 a	74.9 ± 37.2 a	54.0 ± 36.4 a	25.3 ± 10.1 b	3.5 ± 0.9
cotton 2	130 ± 75.1 ab	11.2 ± 4.9	89.6 ±45.1 abc	43.6 ± 18.1 ab	42.4 ± 15.8 ab	29.6 ± 19.9 ab	4.2 ± 1.8
viscose 1 *	277 ± 83.7 a	15.1 ± 5.2	132 ± 43.6 ab	67.4 ± 18.0 a	36.7 ± 10.2 ab	15.3 ± 6.6 b	3.5 ±1.0
viscose 2	52.9 ± 6.2 b	4.3 ± 0.4	26.7 ± 4.8 bc	20.8 ± 3.1 b	12.0 ± 0.8 b	17.8 ± 7.4 b	4.4 ± 1.3
viscose 3	227 ± 195 ab	13.7 ± 9.8	118 ± 102 abc	53.0 ± 38.6 ab	36.0 ± 22.3 ab	13.9 ± 4.0 b	3.3 ± 0.9
*p*	0.087	0.158	0.093	0.042	0.090	0.042	0.619

Values are means of three replicates (*n* = 3) ± standard deviation. * indicates fabrics evaluated in the preliminary trial. Different letters (within columns) indicate significant differences amongst treatments (*p* < 0.05, one-way ANOVA).

**Table 4:** Concentration of glycosylated phenols (µg/kg) detected in Viognier grapes that were enclosed in different fabric coverings (for 72 h) and had been turned inside out following repeated smoke exposure.

	Guaiacol Glycosides	4-Methyl Guaiacol Glycosides	Phenol Glycosides	Cresol Glycosides	Syringol Glycosides	4-Methyl Syringol Glycosides
control	24 ± 0.1 c	2.2 ± 0 c	9.0 ± 0.2 c	12.5 ± 0.3 b	6.7 ± 0.1 c	2.3 ± 0 c
ACF cloth *	227 ± 52 bc	37 ± 10.0 c	71 ± 8 c	107 ± 24.8 b	44 ± 12.3 c	5.3 ± 1.4 c
cotton 1 *	1926 ± 542 a	482 ± 246 a	320 ± 49.2 a	978 ± 264 a	191 ± 31.3 b	20 ± 5.6 b
cotton 2	1375 ± 296 a	381 ± 129 a	257 ± 68.1 ab	923 ± 272 a	352 ± 81.4 a	37 ± 7 a
viscose 1 *	1811 ± 205 a	337 ± 83 ab	315 ± 29.9 a	844 ± 87.7 a	255 ± 13.4 b	40 ± 0.9 a
viscose 2	715 ± 41 b	114 ± 7.5 bc	186 ± 48 b	334 ± 19.2 b	239 ± 30.5 b	33 ± 1.4 a
viscose 3	1811 ± 601 a	363 ± 176 a	224 ± 58.2 b	834 ± 272 a	216 ± 88.5 b	32 ± 16.3 ab
*p*	<0.0001	0.003	<0.0001	<0.0001	<0.0001	<0.0001

Values are means of three replicates (*n* = 3) ± standard deviation. * indicates fabrics evaluated in the preliminary trial. Different letters (within columns) indicate significant differences amongst treatments (*p <* 0.05, one-way ANOVA).

**Table 5:** Comparison of volatile phenol concentrations (µg/kg) in control and smoke-exposed Merlot grapes, with and without bunches being enclosed in different fabric coverings during smoke exposure (where fabrics were either used for the first time or were used after being washed and dried, following repeated smoke exposure).

	Guaiacol	4-Methyl Guaiacol	*o*-Cresol	*m*-Cresol	*p*-Cresol	Syringol	4-Methyl Syringol
control	nd	nd	nd	nd	nd	nd	nd
control *	nd	nd	nd	nd	nd	nd	nd
smoke	18.7 ± 2.4	2.6 ± 0.2	7.0 ± 1.3	6.4 ± 0.8	5.8 ± 0.4	25.1 ± 13.7	9.7 ± 3.0
smoke *	18.4 ± 2.8	3.4 ± 0.2	7.5 ± 0.6	5.5 ± 0.3	6.2 ± 0.4	56.9 ± 18.5	16.2 ± 3.7
*p*	0.811	0.003	0.579	0.263	0.297	0.090	0.096
cotton 1	3.9 ± 1.6	nd	nd	1.5 ± 0.3	1.0 ± 1.7	nd	nd
cotton 1 *	7.3 ±3.1	1.7 ± 0.3	2.4 ± 0.6	2.0 ± 0.3	3.1 ±0.3	nd	nd
*p*	0.131	na	na	<0.001	0.130	na	na
cotton 2	3.8 ± 0.5	nd	nd	1.8 ± 0.5	1.0 ± 1.7	nd	nd
cotton 2 *	7.2 ± 1.6	1.7 ± 0.2	2.9 ± 0.5	2.5 ± 0.5	2.4 ±2.1	nd	nd
*p*	0.051	na	na	0.282	0.332	na	na
viscose 1	10.4 ± 1.8	1.6 ± 0.1	3.2 ± 0.3	2.5 ± 0.3	3.4 ± 0.1	1.3 ± 0.2	nd
viscose 1 *	12.7 ± 2.8	2.6 ± 0.3	4.8 ± 0.6	2.7 ± 0.4	4.4 ± 0.1	2.5 ± 0.8	nd
*p*	0.321	0.025	0.070	0.330	0.033	0.099	na
viscose 2	11.5 ± 1.0	1.8 ± 0.1	3.8 ± 0.3	3.2 ± 0.5	4.1 ± 0.3	4.9 ± 1.5	2.6 ± 0.3
viscose 2 *	16.2 ± 2.0	3.1 ± 0.3	6.2 ± 0.4	3.7 ± 0.8	4.9 ± 0.1	10.7 ± 4.9	4.5 ± 2.1
*p*	0.035	0.020	0.003	0.116	0.045	0.253	0.293
viscose 3	8.8 ± 0.8	1.5 ± 0.2	2.9 ± 0.1	2.7 ± 0.5	3.1 ± 0.2	nd	nd
viscose 3 *	9.5 ± 1.7	2.3 ± 0.1	4.0 ± 0.9	2.5 ± 0.3	3.9 ± 0.3	2.1 ± 0.3	nd
*p*	0.601	0.030	0.177	0.351	0.103	na	na

Values are means of three replicates (*n* = 3) ± standard deviation; nd = not detected. * indicates treatments using fabrics that had been washed and dried following repeated smoke exposure. *p* values are from *t*-tests (*p* < 0.05); na = not applicable.

**Table 6:** Comparison of volatile phenol concentrations (µg/kg) in control and smoke-exposed Merlot grapes, with and without bunches being enclosed in different ACF cloth coverings during smoke exposure.

	Guaiacol	4-Methyl Guaiacol	*o*-Cresol	*m*-Cresol	*p*-Cresol	Syringol	4-Methyl Syringol
control	nd	nd	nd	nd	nd	nd	nd
smoke	18.4 ± 2.8 a	3.4 ± 0.2 a	7.5 ± 0.6 a	5.5 ± 0.3 a	6.2 ± 0.4 a	56.9 ± 18.5 a	16.2 ± 3.7 a
ACF cloth	4.3 ± 1.0 b	1.3 ± 0.1 b	0.9 ± 0.1 b	1.5 ± 0.4 b	1.1 ± 1.9 b	7.8 ± 2.7 b	1.9 ± 0.4 b
ACF (single backing)	nd	nd	nd	nd	nd	1.6 ± 0.2 b	nd
ACF (double backing)	nd	nd	nd	nd	nd	2.1 ± 0.7 b	nd
*p*	<0.0001	<0.0001	<0.0001	<0.0001	0.001	0.001	<0.0001

Values are means of three replicates (*n* = 3) ± standard deviation; nd = not detected. Different letters (within columns) indicate significant differences amongst treatments (*p* < 0.05, one-way ANOVA).

## Data Availability

The original contributions presented in the study are included in the article/Supplementary Material, further inquiries can be directed to the corresponding author.

## Supplementary Materials

The following supporting information can be downloaded at: https://www.mdpi.com/article/10.3390/foods14091550/s1, Figure S1: Photograph of the activated carbon fibre cloth bonded with a non-woven fabric on one side; Table S1: Concentration of volatile phenols (µg/kg) in control and smoke-exposed Viognier grapes, with and without bunches being enclosed in activated carbon fabric (ACF), cotton or viscose coverings during first, second, third, fifth, seventh and tenth replicate smoke treatments; Table S2: Concentration of glycosylated volatile phenols (µg/kg) detected in Viognier grapes that were enclosed in different fabric coverings (for 3 d), after repeated exposure to smoke (10 × 15 min) and being turned inside out.

## Abbreviations

The following abbreviations are used in this manuscript:

ACF	Activated carbon fibre
ANOVA	Analysis of variance
GC-MS	Gas chromatography-mass spectrometry
HPLC-MS/MS	High-performance liquid chromatography-tandem mass spectrometry
SIDA	Stable isotope dilution assay
VOCs	Volatile organic compounds
VPs	Volatile phenols
