# Supplementary material for: Evaluating the Potential for Different Fabrics to Protect Grapes from Contamination by Smoke
Source: Foods. 2025 Apr 28;14(9):1550. doi: 10.3390/foods14091550 (PMC12071767; doi:10.3390/foods14091550)
Supplement: Supplementary file 1 [file foods-14-01550-s001.zip › foods-3591297-supplementary.pdf]

foods

Supplementary Materials

Article

Evaluating the Potential for Different Fabrics to Protect Grapes from Contamination by Smoke

Tingting Shi, Renata Ristic and Kerry Wilkinson *

School of Agriculture, Food and Wine, The University of Adelaide, PMB 1, Glen Osmond, SA 5064, Australia; t.shi@adelaide.edu.au (T.S.); renata.ristic@adelaide.edu.au (R.R.)

* Correspondence: kerry.wilkinson@adelaide.edu.au; Tel: +61-8-8313-7360

Academic Editor: Francisco Artés-Hernández

Received: 1 April 2025
Revised: 24 April 2025
Accepted: 25 April 2025
Published: 28 April 2025

Citation: Shi, T.; Ristic, R.; Wilkinson, K. Evaluating the Potential for Different Fabrics to Protect Grapes from Contamination by Smoke. *Foods* **2025**, *14*, 1550. https://doi.org/10.3390/foods14091550

Copyright: © 2025 by the authors. Licensee MDPI, Basel, Switzerland. This article is an open access article distributed under the terms and conditions of the Creative Commons Attribution (CC BY) license (https://creativecommons.org/licenses/by/4.0/).

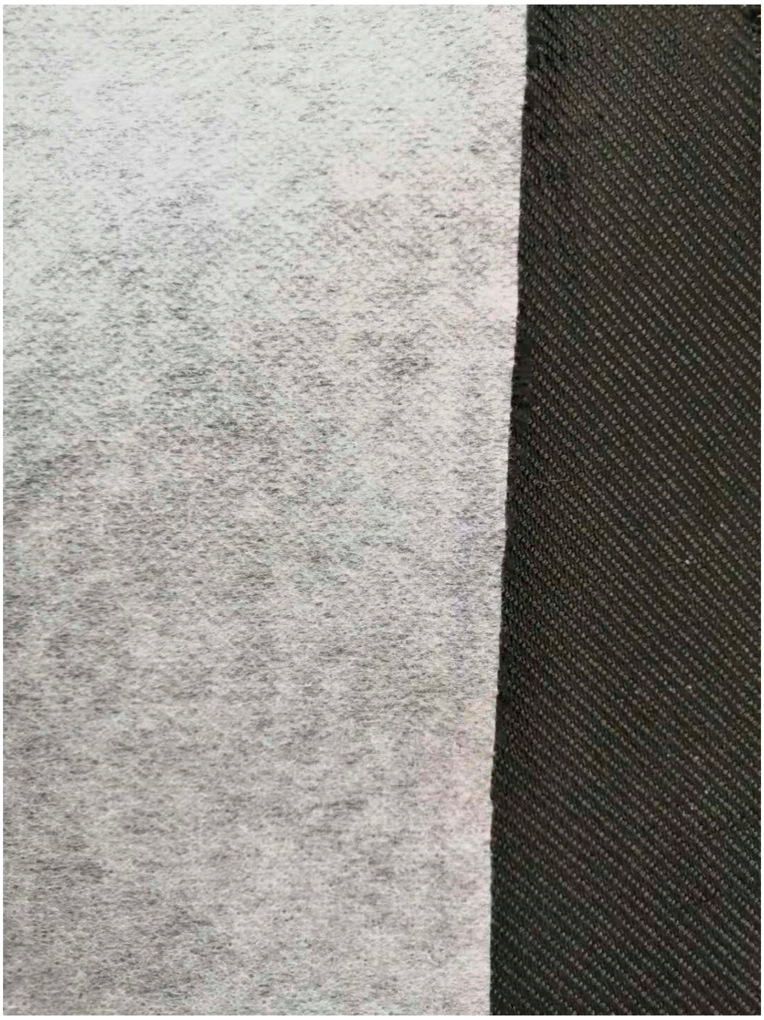

Figure S1. Photograph of the activated carbon fiber cloth bonded with a non-woven fabric on one side.

Table S1. Concentration of volatile phenols (µg/kg) in control and smoke-exposed Viognier grapes, with and without bunches being enclosed in activated carbon fabric (ACF), cotton or viscose coverings during first, second, third, fifth, seventh and tenth replicate smoke treatments.

		Guaiacol	4-Methyl Guaiacol	*o*-Cresol	*m*-Cresol	*p*-Cresol	Syringol	4-Methyl Syringol
	control	nd	nd	nd	nd	nd	nd	nd
smoke 1	smoke	18.7 ± 2.4 a	2.6 ± 0.2 a	7.0 ± 1.3 a	6.4 ± 0.8 a	5.8 ± 0.4 a	25.1 ± 13.7 a	9.7 ± 3.0 a
	ACF*	nd	nd	nd	nd	nd	nd	nd
	cotton 1*	3.9 ± 1.6 d	nd	nd	1.5 ± 0.3 d	1.0 ± 1.7 c	nd	nd
	cotton 2	3.8 ± 0.5 d	nd	nd	1.8 ± 0.5 cd	1.0 ± 1.7 c	nd	nd
	viscose 1*	10.4 ± 1.8 bc	1.6 ± 0.1 b	3.2 ± 0.3 b	2.5 ± 0.3 bcd	3.4 ± 0.2 b	1.3 ± 0.2 b	nd
	viscose 2	11.5 ± 1.0 b	1.8 ± 0.1 b	3.8 ± 0.3 b	3.2 ± 0.5 b	4.1 ± 0.3 b	4.9 ± 1.5 b	2.6 ± 0.3 b
	viscose 3	8.8 ± 0.8 c	1.5 ± 0.2 b	2.9 ± 0.1 b	2.7 ± 0.5 bc	3.1 ± 0.2 b	nd	nd
	p	<0.0001	<0.0001	<0.0001	<0.0001	<0.0001	0.001	<0.0001
smoke 2	smoke	98.1 ± 49.4 a	9.5 ± 3.9 a	40.6 ± 14.9 a	28.0 ± 9.4 a	21.1 ± 6.4 a	18.3 ± 3.8 a	8.9 ± 1.2 a
	ACF*	4.7 ± 1.8 b	1.3 ± 0.2 b	1.5 ± 0.5 b	1.9 ± 0.3 b	3.0 ± 0.1 b	5.6 ± 2.0 c	nd
	cotton 1*	10.7 ± 1.0 b	1.4 ± 0.1 b	3.7 ± 0.1 b	2.9 ± 0.5 b	3.8 ± 0.3 b	nd	nd
	cotton 2	16.2 ± 0.1 b	1.8 ± 0.1 b	3.8 ± 0.2 b	3.2 ± 0.3 b	3.9 ± 0.5 b	nd	nd
	viscose 1*	23.5 ± 5.3 b	3.0 ± 0.5 b	8.3 ± 1.3 b	5.4 ± 0.5 b	6.8 ± 0.5 b	4.4 ± 0.6 cd	1.9 ± 0.1 c
	viscose 2	25.6 ± 4.0 b	2.5 ± 0.3 b	8.4 ± 1.2 b	5.5 ± 1.0 b	6.2 ± 0.5 b	13.8 ± 3.2 b	4.5 ± 0.2 b
	viscose 3	25.9 ± 7.8 b	3.0 ± 0.9 b	9.1 ± 3.2 b	5.0 ± 1.2 b	6.1 ± 1.1 b	1.5 ± 0.5 d	nd
	p	0.001	<0.0001	<0.0001	<0.0001	<0.0001	<0.0001	<0.0001
smoke 3	smoke	416 ± 165 a	33.1 ± 14.4 a	177 ± 68.5 a	108 ± 48.4 a	76.0 ± 31.4 a	111 ± 23.5 a	28.1 ± 4.1 a
	ACF*	6.6 ± 1.6 d	1.4 ± 0.2 c	2.6 ± 0.7 d	2.5 ± 0.4 c	3.4 ± 0.5 c	5.9 ± 1.3 bc	nd
	cotton 1*	63.4 ± 11.1 cd	4.4 ± 0.6 c	19.5 ± 2.8 cd	9.2 ± 1.2 bc	9.0 ± 0.8 c	1.9 ± 0.4 c	nd
	cotton 2	143 ± 62.3 bc	8.7 ± 3.7 bc	43.3 ± 17.6 bcd	18.2 ± 7.6 bc	15.1 ± 5.6 bc	2.1 ± 0.1 c	nd
	viscose 1*	150 ± 50.7 bc	10.5 ± 3.3 bc	50.1 ± 18.2 bc	21.4 ± 6.7 bc	21.8 ± 6.5 bc	11.5 ± 2.8 bc	3.5 ± 0.6 c
	viscose 2	232 ± 69.5 b	16.7 ± 4.2 b	80.4 ± 21.1 b	38.2 ± 9.5 b	33.2 ± 8.1 b	21.2 ± 7.1 b	7.6 ± 2.0 b
	viscose 3	126 ± 17.4 bc	8.6 ± 1.2 bc	43.4 ± 5.3 bcd	19.1 ± 2.7 bc	18.4 ± 1.3 bc	2.9 ± 0.5 c	1.0 ± 0.9 c
	p	<0.0001	<0.0001	<0.0001	<0.0001	<0.0001	<0.0001	<0.0001
smoke 5	smoke	1108 ± 275 a	92.2 ± 24.4 a	352 ± 69.7 a	198 ± 39.1 a	162 ± 32.7 a	229 ± 67.6 a	73.8 ± 15.4 a
	ACF*	10.9 ± 6.5 c	1.8 ± 0.4 c	3.5 ± 1.8 c	3.3 ± 1.2 c	3.9 ± 0.5 c	14.2 ± 7.3 c	3.1 ± 1.2 c
	cotton 1*	178 ± 44.5 bc	9.7 ± 2.0 bc	60.0 ± 17.0 bc	23.5 ± 5.9 c	23.1 ± 5.6 bc	3.7 ± 0.6 c	1.0 ± 0.9 c
	cotton 2	227 ± 19.8 bc	13.9 ± 0.8 bc	70.9 ± 7.8 b	32.2 ± 3.4 bc	26.3 ± 2.1 bc	9.5 ± 3.6 c	1.7 ± 0.2 c
	viscose 1*	200 ± 36.2 bc	13.9 ± 2.5 bc	65.9 ± 9.4 bc	28.8 ± 4.3 bc	27.0 ± 2.6 bc	12.5 ± 3.0 c	5.7 ± 0.9 c
	viscose 2	358 ± 154 b	29.2 ± 13.0 b	114 ± 50.3 b	62.2 ± 29.8 b	51.7 ± 21.7 b	83.7 ± 11.6 b	27.5 ± 3.3 b
	viscose 3	170 ± 35.7 bc	10.5 ± 1.4 bc	58.7 ± 16.3 bc	24.7 ± 6.4 c	24.6 ± 5.7 bc	3.2 ± 1.5 c	1.8 ± 0.4 c
	p	<0.0001	<0.0001	<0.0001	<0.0001	<0.0001	<0.0001	<0.0001
smoke 7	smoke	476 ± 103 a	35.1 ± 5.7 a	182 ± 22.6 a	70.8 ± 7.1 a	74.9 ± 9.5 a	82.3 ± 49.7 a	34.8 ± 15.2 a
	ACF*	7.0 ± 3.4 e	1.7 ± 0.5 e	2.2 ± 1.3 e	3.4 ± 2.2 d	2.5 ± 2.2 d	12.1 ± 7.2 b	1.8 ± 1.5 c
	cotton 1*	122 ± 30.5 de	8.2 ± 2.0 de	43.1 ± 13.0 d	17.9 ± 4.7 c	19.0 ± 4.9 c	5.0 ± 2.8 b	1.9 ± 0.7 c
	cotton 2	279 ± 88.3 bc	15.3 ± 4.8 cd	84.9 ± 27.7 c	26.8 ± 4.9 c	31.9 ± 10.3 bc	11.1 ± 4.4 b	2.5 ± 0.9 c
	viscose 1*	242 ± 89.4 bcd	17.1 ± 6.6 bc	80.6 ± 27.5 c	29.3 ± 9.5 c	32.4 ± 10.8 bc	13.6 ± 4.9 b	6.6 ± 2.7 c
	viscose 2	351 ± 43.1 b	24.0 ± 5.2 b	121 ± 17.9 b	43.9 ± 13.3 b	45.4 ± 9.7 b	42.1 ± 24.9 b	20.7 ± 9.2 b
	viscose 3	199 ± 30.9 cd	14.0 ± 2.3 cd	67.0 ± 9.9 cd	25.3 ± 4.2 c	28.1 ± 4.8 c	12.2 ± 4.7 b	3.9 ± 1.0 c
	p	<0.0001	<0.0001	<0.0001	<0.0001	<0.0001	0.007	<0.0001

smoke 10	smoke	399 ± 76.1 a	28.0 ± 5.2 a	130 ± 27.1 a	48.2 ± 10.2 a	52.8 ± 10.5 a	67.1 ± 22.6 a	21.7 ± 6.7 a
	ACF*	4.4 ± 1.8 c	1.2 ± 0.1 d	1.2 ± 0.3 c	1.6 ± 0.3 d	1.0 ± 1.8 c	3.7 ± 0.2 b	nd
	cotton 1*	170 ± 65.5 b	9.6 ± 2.6 bc	47.1 ± 13.3 b	15.8 ± 3.8 c	20.1 ± 5.3 b	10.6 ± 7.1 b	2.6 ± 1.1 c
	cotton 2	128 ± 32.0 b	9.5 ± 1.9 bc	42.0 ± 6.4 b	19.2 ± 2.0 bc	18.3 ± 2.6 b	13.0 ± 4.1 b	3.2 ± 0.7 bc
	viscose 1*	159 ± 42.1 b	10.7 ± 3.2 bc	47.9 ± 13.0 b	19.0 ± 6.3 bc	19.4 ± 5.1 b	20.9 ± 7.1 b	7.4 ± 1.4 bc
	viscose 2	123 ± 41.0 b	8.0 ± 2.2 c	44.4 ± 12.5 b	15.5 ± 3.3 c	17.7 ± 4.9 b	16.4 ± 5.1 b	8.1 ± 1.9 b
	viscose 3	211 ± 53.6 b	14.5 ± 3.4 b	64.9 ± 13.6 b	27.7 ± 6.8 b	26.3 ± 4.8 b	16.1 ± 8.3 b	4.3 ± 1.3 bc
	p	<0.0001	<0.0001	<0.0001	<0.0001	<0.0001	<0.0001	<0.0001

Values are means of three replicates ($n = 3$) ± standard deviation; nd = not detected. * denotes fabrics evaluated in preliminary trial. Different letters (within columns, by replicate smoke treatment) indicate significant differences amongst treatments ($p < 0.05$, one-way ANOVA).

Table S2. Concentration of glycosylated volatile phenols (µg/kg) detected in Viognier grapes that were enclosed in different fabric coverings (for 3 d), following repeated exposure to smoke (10 x 15 min) and being turned inside out.

	GuG	GuGG	GuPG	GuR	4MGuG	4MGuGG	4MGuPG	4MGuR	PhPG	PhR	CrG	CrGG	CrPG	CrR	SyrG	SyrGG	SyrPG	MSyrGG	MSyrPG
control	nd	1.2 b	23 d	nd	nd	nd	2.2 c	nd	9.0 c	nd	nd	nd	11.1 c	1.5 d	nd	1.4 c	5.3 c	nd	2.3 c
ACF cloth*	13.7 c	31 b	180 d	1.4 c	2.5 c	9.7 c	24 c	nd	70 c	nd	6.3 b	2.8 b	194 bc	3.4 cd	3.6 c	33 c	7.8 c	2.6 c	2.6 c
cotton 1*	306 a	484 a	1,116 a	19.7 ab	62 a	168 a	239 a	14.0 a	311 a	8.1 ab	147 a	46 a	740 a	45 ab	32 b	145 b	14.3 b	16.5 b	2.9 bc
cotton 2	188 ab	344 a	827 b	15.7 ab	42 abc	125 a	201 a	13.5 a	248 ab	8.4 ab	129 a	44 a	697 a	54 a	58 a	273 a	21 a	32 a	3.9 ab
viscose 1*	284 a	443 a	1,062 ab	22 a	36 abc	107 ab	181 a	12.0 a	302 a	11.2 a	134 a	38 a	623 a	48 ab	49 ab	187 b	19.3 ab	34 a	3.9 ab
viscose 2	69 bc	113 b	523 c	8.8 bc	10 bc	29 bc	69 bc	6.3 b	181 b	5.7 b	38 b	11.1 b	263 b	23 bcd	46 ab	173 b	20 a	27 ab	4.1 a
viscose 3	297 a	500 a	999 ab	14.6 ab	49 ab	143 a	162 ab	8.7 a	218 b	5.2 b	124 a	45 a	636 a	29 abc	39 ab	163 b	14.4 ab	27 ab	3.4 abc
p	0.005	< 0.001	< 0.001	0.005	0.038	0.005	0.002	0.01	< 0.001	0.001	0.002	0.001	< 0.001	0.004	< 0.001	< 0.001	< 0.001	< 0.001	0.019

Values are means of three replicates (n = 3). * indicates fabrics evaluated in the preliminary trial. Different letters (within columns) indicate significant differences amongst treatments ($p < 0.05$, one-way ANOVA). Gu = guaiacol; 4MG = 4-methylguaiacol; Ph = phenol; Cr = cresol; Syr = syringol; MSyr = 4-methylsyringol; G = glucoside; GG = glucose-glucoside; PG = pentose-glucoside; R = rutinoside. PhG, PhGG and 4MSyr were detected at ≤ 2.0 µg/kg in any samples.

Disclaimer/Publisher's Note: The statements, opinions and data contained in all publications are solely those of the individual author(s) and contributor(s) and not of MDPI and/or the editor(s). MDPI and/or the editor(s) disclaim responsibility for any injury to people or property resulting from any ideas, methods, instructions or products referred to in the content.
